# MRI and Pulmonary Function Tests’ Results as Ventilation Inhomogeneity Markers in Children and Adolescents with Cystic Fibrosis

**DOI:** 10.3390/jcm12155136

**Published:** 2023-08-05

**Authors:** Irena Wojsyk-Banaszak, Barbara Więckowska, Aleksandra Szczepankiewicz, Zuzanna Stachowiak, Marta Andrzejewska, Jerzy Juchnowicz, Maciej Kycler, Paulina Famulska, Marta Osińska, Katarzyna Jończyk-Potoczna

**Affiliations:** 1 Department of Paediatric Pulmonology, Allergy and Clinical Immunology, Poznan University of Medical Sciences, 60-572 Poznań, Poland; marta.andrzejewska.rm@gmail.com (M.A.); maciejkycler@gmail.com (M.K.); 2 Department of Computer Science and Statistics, Poznan University of Medical Sciences, 61-701 Poznań, Poland; b.wieckowska@ump.edu.pl (B.W.); jurekj23@wp.pl (J.J.); 3 Molecular and Cell Biology Unit, Poznan University of Medical Sciences, 60-572 Poznań, Poland; alszczep@ump.edu.pl (A.S.); zuzastachowiak9@gmail.com (Z.S.); 4 Pediatric and Cystic Fibrosis Department, Pediatric Hospital in Gdańsk, 80-308 Gdańsk, Poland; p.famulska@gmail.com (P.F.); moononoom.93@gmail.com (M.O.); 5 Department of Paediatric Radiology, Poznan University of Medical Sciences, 61-701 Poznań, Poland; potocznak@op.pl

**Keywords:** cystic fibrosis, children, MRI, pulmonary function tests

## Abstract

Magnetic resonance imaging (MRI) of the chest is becoming more available in the detection and monitoring of early changes in lung function and structure in patients with cystic fibrosis (CF). The aim of this study was to assess the relationship between pulmonary function tests (PFT) and perfusion deficits in CF children measured by MRI. We performed a retrospective analysis of the perfusion lung MRI scans and the results of spirometry, oscillometry, body plethysmography, single-breath carbon monoxide uptake, and multiple-breath washout technique (MBW). There were statistically significant correlations between the MRI perfusion scores and MBW parameters (2.5% LCI, M1/M0, M2/M0), spirometry parameters (FEV_1_, FVC, FEF25/75), reactance indices in impulse oscillometry (X5Hz, X10Hz), total lung capacity (TLC) measured in single breath carbon monoxide uptake, markers of air-trapping in body plethysmography (RV, RV/TLC), and the diffusing capacity of the lungs for carbon monoxide. We also observed significant differences in the aforementioned PFT variables between the patient groups divided based on perfusion scores. We noted a correlation between markers of functional lung deficits measured by the MRI and PFTs in CF children. MRI perfusion abnormalities were reflected sooner in the course of the disease than PFT abnormalities.

## 1. Introduction

Cystic fibrosis is an autosomal recessive disorder caused by a mutation in the *CFTR* (ang. *Cystic Fibrosis Transmembrane Regulator*) gene, encoding a chloride channel that is present in the epithelial cells of many body organs, including the airways [1]. The damage to the lungs in the course of CF may temporarily develop with no clinical manifestations, and therefore sensitive diagnostic tools are particularly important to implement the treatment soon enough to prevent or at least slow the process of irreversible damage [2,3]. 

CF lung disease starts in the small airways with a diameter of less than 2 mm that remains beyond the limit of HRCT or MR image resolution. CT enables the visualization of healthy bronchi down to the eighth generation and MR down to the third [4]. Small airways can therefore be assessed only indirectly with techniques evaluating hyperinflation, air trapping, or inhomogeneity of ventilation or perfusion either in radiological studies or pulmonary function tests [5]. 

Magnetic resonance imaging (MRI) of the chest is becoming more available and popular in the detection and monitoring of early changes in lung structure and function, as well as routine follow-up care in people with cystic fibrosis (CF) [6,7,8]. This radiation-free technique seems particularly feasible for children and adolescents who are much more prone than adults to the adverse effects of radiation. MRI can be successfully performed without sedation in most children aged 5 to 7 years and some even as young as 3 years [9]. In the absence of morphological changes, lung parenchyma shows normal perfusion, while damaged lung segments show impaired perfusion [10]. Perfusion abnormalities might reflect ventilatory defects that arise due to, e.g., mucus plugging of the small and large airways, irrespective of the disease severity, and may become apparent sooner than morphological changes [6,10]. 

In addition to radiological studies in order to assess pulmonary disease in CF, researchers and clinicians utilize pulmonary function tests (PFT). Due to the progress in the standard care in CF, increasing numbers of affected individuals have normal spirometry, and [11] and in this group of patients, the multiple breath washout technique (MBW) is a more sensitive technique [3]. MBW reflects ventilation heterogeneity that provides information on the involvement of the small airways. It has been shown to correlate with lung structural changes as well as the ventilation deficits measured in Hyperpolarized ^129^Xe Magnetic Resonance [3,12]. MBW, however, seems to perform sub-optimally in investigating the treatment response as well as in monitoring patients with more severe lung disease [3,13]. There also remains a group of patients with normal PFT results in whom lung disease might already be present [14]. Therefore, we simultaneously looked at other pulmonary function tests investigating peripheral airways, including impulse oscillometry as well as air trapping indices in body plethysmography and in the diffusing capacity of the lungs for carbon monoxide. 

The objective of this study was to assess the relationship between different PFT results reflecting ventilation deficits and small airway involvement and perfusion deficits measured by MRI in children with CF. Finding the test that would exhibit the best correlation with MRI results might assist in monitoring the patients and planning further control studies.

Preliminary results of this study have been previously reported in the form of an abstract [15].

## 2. Materials and Methods

We conducted a retrospective analysis of lung MRI, spirometry, impulse oscillometry (IOS), body plethysmography, single-breath carbon monoxide uptake (SBCO), and MBW results performed during annual check-up visits in a tertiary academic hospital from 28 May 2017 until 13 October 2021 in pediatric patients with CF. We included all patients who had MRIs performed during their annual review hospitalization. CF was diagnosed based on two positive sweat chloride results and the confirmation of two CF-causing mutations. Follow-up visits are scheduled every three months in our center, and once a year, patients undergo a more comprehensive evaluation, including radiological studies, i.e., MRI for those who cooperate and can perform the study without sedation. During this annual review visit, we also performed very detailed pulmonary function investigations, including oscillometry, body plethysmography, and DLCO. The reason for this approach is to have a wider insight into different lung compartments in our patients. With impulse oscillometry, we find it very informative in younger patients who cannot perform spirometry as well as those with severe lung disease in whom LCI is quite difficult to perform. Most of the patients were in stable conditions, but some of them were diagnosed on admission with exacerbation. 

Pulmonary exacerbation (PE) was diagnosed by the treating physician and verified retrospectively by a specialist in pediatric pulmonology according to EuroCare CF working group criteria [16].

The Bioethics Committee of Poznań University of Medical Sciences approved the study (document KB-990/22). The participants and their caregivers gave their consent for the studies.

### 2.1. Magnetic Resonance

**Figure 1:**
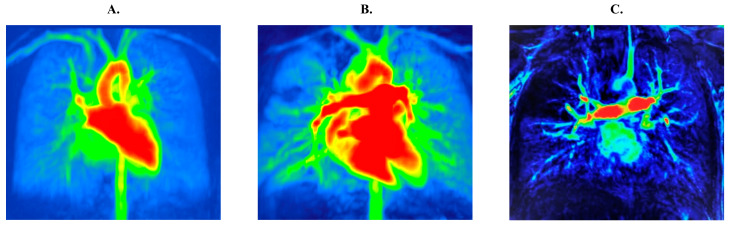
MRI images visualizing the perfusion scale: MRI score 0, no perfusion abnormalities (**A**); MRI score 1–3, perfusion abnormalities comprising less than 50% of the lobe volume (**B**) and MRI score > 3, perfusion abnormalities comprising more than 50% of the lobe volume (**C**).

During the first 48 h after admission PFTs and gadolin, contrast-enhanced MRI of the chest (3T Magnetom Spectra apparatus (Siemens Healthcare Germany, Erlangen, Germany)) was performed. Perfusion abnormalities in the MRI were scored by a board-certified specialist in radiology with an experience with cystic fibrosis following a previous description by Eichinger and colleagues separately for each right and left lobe with 0 as no perfusion abnormalities, 1—perfusion abnormalities comprising less than 50% of the lobe volume and 2—perfusion abnormalities comprising more than 50% of the lobe volume (Figure 1) [17]. The maximum achievable score (MRI perfusion score MRI-PS) was 12. The structural sequences had been performed but were not included in the analysis as the aim of the study was to compare ventilation and perfusion deficits. 

### 2.2. Pulmonary Function Tests

All children performed SBCO, followed by MBW, then by IOS, and then spirometry and body plethysmography. Quality control was performed by a qualified operator experienced in PFT in children and verified by one of the researchers. Spirometry and lung volume measurements (SBCO, body plethysmography) were performed using Lungtest 1000 MES, Poland system, according to the ATS/ERS guidelines for lung function testing, with Global Lung Initiative reference equations [18,19,20,21,22]. The updated technical standards for spirometry were published in 2019, and all studies since January 2020 were performed accordingly. For the statistical analysis, the best of two (TLCO) and three (body plethysmography, spirometry) acceptable maneuvers were used.

Impulse oscillometry parameters included reactance at 5 and 10 Hz (X5Hz and X10Hz, respectively), the area of reactance (AX), and the resonance frequency (Fres). Reactance parameters and AX and Fres provide information on elastic properties of the respiratory system and indirectly on the small airways, where CF lung disease originates. IOS was performed using the Jaeger Vyntus IOS (CareFusion, Hoechberg, Germany) oscillometer according to the ERS technical standards [23]. For the statistical analysis, the best result out of three acceptable maneuvers was used. 

Nitrogen multiple-breath washout tests were performed with an Exhalyzer-D with a mouthpiece (EcoMedics AG, Dürnten, Switzerland) with the reference values of Kentgens et al. in accordance with ERS/ATS consensus statement guidelines [24,25]. The parameters measured included the lung clearance index (LCI) and moment ratios (M1/M0, M2/M0). The higher these parameters are, the lower homogeneity of lung ventilation. M2/M0 especially reflects the washout in the worst ventilated lung units [26]. All results were expressed as the mean of a minimum of two technically acceptable results obtained during one session. We recalculated all the LCI values obtained before 2022 with the Exhalyzer D software (version Spiroware 3.3.1) using Kentgens’s reference values. The change in software was triggered by the reports that identified the error in the nitrogen concentration calculated by the previously used software [27].

For all the pulmonary function tests results, z-score values ≤ 1.64 and ≥1.64 were considered normal.

### 2.3. Statistical Analysis

Statistical analysis was performed with PQStat 1.8.2 and Statistica software (version 12; StatSoft). Data distribution was assessed using the Shapiro–Wilk test. For the comparison of PFT results between patients with different perfusion abnormalities, we used the Kruskal–Wallis test for continuous data and the chi-square test for categorical data. Since this was a real-life study in patients with a broad spectrum of the severity of bronchopulmonary disease, we did not remove the outliers. As a post-hoc test for the Kruskal–Wallis, we used the Dunn–Bonferroni procedure, and for multiple comparisons after the chi-square test, we used the chi-square test with the Benjamini–Hochberg correction. The Z test of significance for Cohen’s kappa was used to assess the agreement for the MRI-PS and PFT results. The cut-off 0.21–0.4 signified fair agreement, 0.41–0.6—moderate agreement, 0.61–0.8 good agreement, and >0.8 very good agreement. Spearman correlation coefficients were calculated with Bonferroni correction for multiple comparisons; the *p*-value of <0.05 is considered statistically significant.

## 3. Results

We collected the data from 87 consecutive visits of 39 patients, their median age being 12 years (range 6–18). The characteristic of the patients is given in Table 1. There were 87 spirometry measurements, 71 MBW (on 16 occasions, patients did not perform MBW due to a severe cough), 85 IOS measurements (due to technical problems with the device), 82 body plethysmography measurements (on five occasions, patients refused to be locked in the cabin) and 54 SBCO examinations (due to technical problems with the device and inability of patients to perform the test). The mean time span between the visits was 12 months. A total of 8 patients underwent 1 MRI study, 18 patients 2 MRI studies, 9 patients 3, and 4 patients 4 MRI during the study period.

In 65% of measurements, the patients had abnormal LCI, and on 29% of occasions, patients had decreased FEV_1_. Abnormal IOS parameters (X5Hz, X10Hz, Fres) were seen during 1%, 6%, and 85% of visits, respectively. Restriction and hyperinflation were seen in 4% and 78% of body plethysmography examinations, respectively, and in 13% and 37% of SBCO examinations (Table 2). There were no perfusion deficits in MRI in 41% of examinations (Table 3).

During 39% of all the visits, when the MRI-PS was 0, patients had increased LCI, and on 32% of visits, patients with normal LCI had MRI-PS > 1. During 48.4% and 55.1% visits during which patients had normal FEV_1_ and FVC, respectively, their MRI-PS were increased. On 61.9% and 60% of occasions, respectively, patients with normal X5Hz and X10Hz results had increased MRI-PS. A normal total lung capacity (TLC) measured in body plethysmography was accompanied on 69.2% of occasions by an increased MRI-PS, and patients who had no hyperinflation in body plethysmography on 13.3% of occasions had increased MRI-PS. The highest concordance for the MRI-PS and PFT results were seen for LCI, moments ratios, and the RV/TLC ratio in body plethysmography (Table 2).

We found statistically significant correlations between MRI-PS and MBW, spirometry parameters, X5Hz and X10Hz, TLC in SBCO, markers of air-trapping in body plethysmography (RV and RV/TLC), and the TLCO (Table 4).

**Figure 2:**
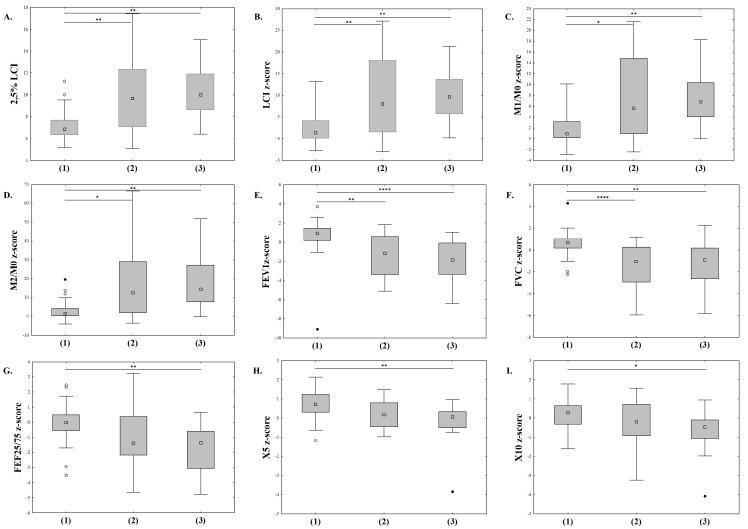
Differences in selected PFT parameters between three groups of patients distinguished by MRI classification: 2.5% LCI, F(2;68) = 10.386, and *p* < 0.001 (**A**); LCI z-score, F(2;6) = 10.376, and *p* < 0.001 (**B**); M1/M0 z-score, F(2;68) = 10.118, and *p* < 0.001 (**C**); M2/M0 z-score, F(2;68) = 8.41, and *p* < 0.001 (**D**); FEV_1_z-score, F(2;84) = 13.65, and *p* < 0.001 (**E**); FVC z-score, F(2;84) = 16.25, and *p* < 0.001 (**F**); FEF25/75 z-score, F(2;84) = 8.309, and *p* < 0.001 (**G**); X5 z-score, F(2;82) = 8.21, and *p* < 0.001 (**H**); X10 z-score, F(2;82) = 4.15, and *p* = 0.019 (**I**). Groups by MRI classification: no perfusion deficits (1), mild perfusion deficits (2), severe perfusion deficits (3). Analysis was performed using the Kruskal–Wallis one-way ANOVA; (○) outliers, (●) extreme outliers. Pairwise comparison with Dunn–Bonferroni test: *p* ≤ 0.05 (*); *p* ≤ 0.01 (**); *p* ≤ 0.0001 (****).

**Figure 3:**
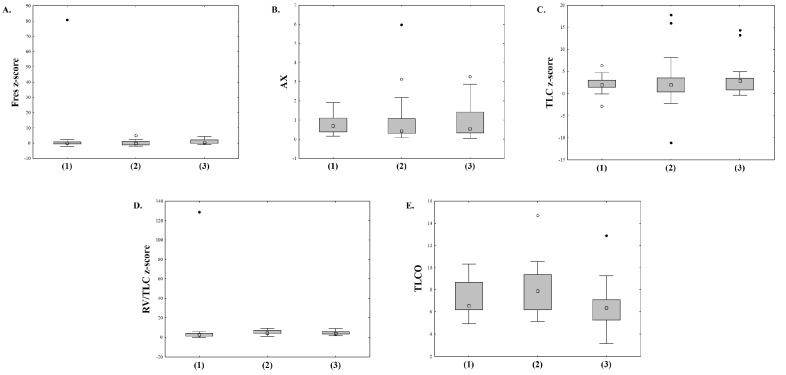
Differences in selected PFT parameters between three groups of patients distinguished by MRI classification: Fres z-score, F(2;82) = 0.546 and *p* = 0.581 (**A**); AX, F(2;82) = 0.309 and *p* = 0.735 (**B**); TLC z-score, F(2;74) = 0.668 and *p* = 0.516 (**C**); RV/TLC z-score, F(2;75) = 0.104, and *p* = 0.902 (**D**); SBCO TLCO, F(2;51) = 2.074 and *p* = 0.136 (**E**). Groups by MRI classification: no perfusion deficits (1), mild perfusion deficits (2), severe perfusion deficits (3). Analysis was performed using the Kruskal–Wallis one-way ANOVA; (○) outliers, (●) extreme outliers.

We then divided the patients into three groups based on perfusion deficits: no perfusion deficits (MRI-PS 0), mild perfusion deficits (MRI-PS 1–3), and severe perfusion deficits (MRI-PS > 3). The characteristics of patients in each group are given in Table 3. We found significant differences between the groups in all MBW and spirometry parameters, as well as reactance indices in IOS (Figure 2). The differences for body plethysmography (except for RV), SBCO parameters, and TLCO did not reach statistical significance (Figure 3).

## 4. Discussion

To our knowledge, this is the first study comparing such a wide array of concurrently performed PFTs and MRI perfusion scores. In our study, perfusion deficits visualized in MRI correlated with indices of ventilation abnormalities derived from spirometry, body plethysmography, IOS, MBW, as well as SBCO. The highest correlation of MR-PS was seen for spirometry indices and LCI. In many patients with normal PFT results, perfusion deficits in MRI were present. 

MRI is comparable to CT in the structural assessment of morphological changes in CF lung disease and is superior with regard to pulmonary perfusion [17,28,29]. Perfusion deficits result from the reflex of hypoxic vasoconstriction or tissue destruction and have been described as an indicator of mucus plugging and airway obstruction in children with CF, irrespective of lung disease severity [6,30]. Perfusion defects correlate with ventilation abnormalities in MRI-derived oxygen-enhanced T_1_ relaxation measurements and ventilation—weighted Fourier decomposition MRI with dynamic contrast-enhanced (DCE) MRI [31]. It has been reported that perfusion abnormalities might be present in the absence of morphological changes in MRI, and over 80% of children in the first six years of life present with abnormal lung perfusion [6]. Additionally, MRI shows the regional distribution of perfusion deficits, and these qualities make this a superior method over more commonly used PFTs [10]. In our study of children and adolescents with CF, perfusion deficits were present during 59% of visits. Higher MRI-PS were present in older patients, patients with pancreatic insufficiency, and during exacerbations. There were no differences in MRI-PS with regard to genotype, BMI, or chronic infection status. In a study conducted by Stahl and colleagues, MRI perfusion deficits were more common (77% of children), and their incidence increased with age [32]. More patients in their group were tested during exacerbation (35% vs. 25% in our group), and more had elevated LCI (85% vs. 72% in our group), which might partly explain these results. 

In our study, during 11.3%, 14.1%, and 11.3% of visits, children had normal results of the MBW test (LCI, M1/M0, and M2/M0, respectively) and an increased MRI-PS. This is less frequent than Stahl and colleagues found in their study, in which 25% of children with normal LCI had evidence of mucus plugging in MRI; however, as mentioned above, these children might have presented with more severe lung disease [32]. On the other hand, during 14% of visits, patients with an MRI-PS of 0 had increased LCI. We found statistically significant, albeit fair to moderate, correlations between MRI-PS and MBW parameters (r = 0.506 for LCI z-score, 0.49 for M1/M0, and 0.35 for M2/M0). In the above-mentioned study by Stahl et al., LCI correlated with abnormal perfusion in MRI (r = 0.74) [32]. These values, however, cannot be directly compared since we used newer LCI software that recalculated the LCI values since an error in the previously applied software had been discovered. The groups of children distinguished based on their MRI-PS had statistically different LCI and moment ratios (Figure 2). Moment ratios can detect ventilation inhomogeneity in the most peripheral parts of the lung at the latter portion of a washout than LCI, with M2/M0 being more sensitive to clinical deterioration than other MBW parameters [12,26]. The discordance between the results of MRI-PS and MBW results shows that the methods are complementary. There remains a group of patients in whom MRI would still provide information on their lung status in addition to MBW results that might change the therapeutic plan.

The results were much more discordant for MRI-PS and spirometry indices, with normal results of FEV_1_ in 34% of cases, FVC in 44%, and FEF_25/75_ in 36% of cases with increased MRI-PS. These results are not surprising and corroborate the better diagnostic value of LCI compared to spirometry in patients with mild lung disease. In their longitudinal study, Bakker et al. showed that 70% of CF patients at the age of 8 had FVC values over the 10th percentile and 50% FEV_1_ values in the same range [33]. FEF_25/75_ values reflect more accurately the obstruction of small airways than FEV_1_ [34]; however, in our study, it was not superior to other spirometry indices. The discordant results of spirometry and MRI were much more frequent than observed with MBW parameters or hyperinflation measures from body plethysmography and SBCO and were similar to IOS reactance indices. In most cases of abnormal spirometry results, perfusion deficits were present in MRI. We found statistically significant differences between all spirometry indices in patients with no perfusion abnormalities versus mild and more severe deficits (Figure 2). The correlation coefficients between both FEV_1_ and FVC, but not FEF_25/75_ and MRI-PS, were slightly higher than in the case of the MBW results. It might be due to the fact that we included children along the total spectrum of lung involvement from mild to severe. We did not find a study comparing MRI-PS deficits and spirometry results in cystic fibrosis. Smith et al., however, found a strong correlation between FEV_1_ and ventilation deficits measured with hyperpolarised helium-3 MRI which might, in fact, reflect similar pathophysiology [35]. The same group, however, did not find any correlation between FEV_1_ and ventilation defect percentage (VDP) measured by ^129^Xe MRI [8].

The concordance of abnormal results between IOS indices and MRI-PS was poor. On more than half of all occasions, patients had abnormal MRI-PS and reactance, as well as Fres values within normal limits. Most patients in our study had IOS results within normal limits, though, and all patients with MRI-PS 0 had normal reactance values. Both X5HZ and X10HZ, but neither Fres nor AX, correlated poorly, albeit statistically significantly with MRI-PS. The reactance values also varied depending on perfusion deficits in MRI (Figure 2 and Figure 3). Low-frequency reactance, as well as Fres, are measures of elastic properties of the lung parenchyma and peripheral airways and are influenced by mucus plugging and, consequently, ventilation deficits [36]. Abnormal reactance indices, though rarely seen in our patients, similar to abnormal spirometry parameters, were inadvertently associated with the presence of perfusion deficits in MRI.

Air trapping is widely present in CF pediatric patients and was reported in 58% of infants up to 94% of school-aged children [12]. Unfortunately, the sensitivity of LCI to detect air trapping was 38%, so other complementary methods should be utilized [12]. Air trapping indices measured with body plethysmography are sensitive, although infrequently used, and probably underestimated markers of lung disease progression in CF [37]. Hyperinflation was present in 39% of patients aged between 6–8 years and up to 67% at 18 years of age [37]. In our study, hyperinflation determined by increased RV/TLC ratio was present during 81% of visits in body plethysmography and 63% in SBCO (Table 3). The discrepancy between RV and TLC measured by these two methods is due to the different measurement techniques. In body plethysmography, lung volumes are calculated from the pressure changes at the mouth, and with SBCO, the amount of air that is exchanged during a single breath is measured. With unventilated lung segments blocked with mucus or atelectatic, the actual lung volumes measured by the gas exchange during a single breath might be underestimated.

Deficits of perfusion in MRI were seen in 69% of patients with normal TLC results in body plethysmography and on 73% of occasions with normal TLC results in SBCO. Much higher concordance was seen in the negative test results: the lack of hyperinflation was accompanied by abnormal MRI-PS in 13% (body plethysmography) and 45% (SBCO) of cases. It seems quite unlikely that a patient with a normal RV/TLC ratio in body plethysmography would have perfusion deficits in MRI. 

There were mild correlations between RV and RV/TLC measured in body plethysmography, TLC measured in SBCO, and MRI-PS. We found no studies comparing MRI-PS with TLC or RV. In a study of functional MRI without contrast using matrix pencil decomposition, Nyilas et al. showed a strong correlation between the index of perfusion impairment (RQ) and RV/TLC [38]. There was also a correlation described between ventilation abnormalities in ^3^He-MRI and RV/TLC [35], although the same group, as mentioned above, did not find the correlation between changes in ventilation deficit indices measured with ^129^Xe -MRI and RV/TLC [8]. In this study ^129^Xe -MRI was shown to be superior to body plethysmography (as well as spirometry and LCI) in tracking the progression of pulmonary disease [8]. Air trapping indices from body plethysmography correlated better with MRI-PS, probably because of the different measurement methods (as described above). The correlation between TLC measured by SBCO and not by body plethysmography and MRI-PS might result from the fact that body plethysmography could overestimate lung capacity by the amount of air present in extrathoracic body compartments. 

In half of the cases, when TLCO results did not indicate dysfunction of capillary-alveolar gas transfer, MRI showed perfusion deficits. However, similarly to spirometry and IOS results, in most cases of diminished TLCO, abnormal perfusion was present in MRI. 

The advantages of our study include the application of a wide array of complementary PFTs and their association with the MRI-PS. We tested a group of children with a broad spectrum of disease severity in a naturalistic clinical setting. Despite the retrospective nature of our study, the PFTs and MRI were performed over a period not exceeding 48 h, which minimizes the risk of potential changes in lung status in the time period between the tests. 

Among the disadvantages, we need to mention the retrospective nature of our study. Patients were recruited from one center only. We did not include preschool children and infants as they would need sedation for the MRI, and most PFTs would not be possible to perform in this age group. The broad spectrum of disease severity in our patients, and consequently, the distribution of the pulmonary function tests data, might have skewed the results. Only 40% of our patients were diagnosed as a consequence of newborn screening that had been introduced in our country in 2010. Also, at the time of the study, the CFTR modulators had not been reimbursed in Poland; therefore, no patient in the study received these drugs. These two facts might limit the applicability of the results in populations diagnosed soon after birth (most children nowadays) and in patients receiving highly effective modulator treatments (HEMT). Nevertheless, there are still many people with cystic fibrosis who had been diagnosed after the neonatal period, and many still do not receive HEMT due to ineligibility, side effects, lack of reimbursement, etc. Additionally, during the period of data collection (2019), an update in spirometry technical standards was published, and we changed our practice in 2020 accordingly. This should not have affected the results, however, as the only change that had been introduced was the lack of FET criterium in the new standards, so the updated criteria are less stringent ones. Other technical standards had already been introduced in our Pulmonary Function Test Laboratory before the update. Last but not least, MRI does not involve ionizing radiation; however, in order to achieve perfusion scans, patients are infused with contrast medium (gadolin), which poses a risk of adverse events. The emerging novel techniques: oxygen-enhanced MRI, Fourier decomposition MRI, and phase-resolved functional lung MRI (PREFUL) might provide us in the future with safer and equally effective diagnostic modalities for monitoring bronchopulmonary disease in people with CF [39].

## 5. Conclusions and Possible Clinical Applications

Our results strongly support the use of MRI as a promising outcome measure to detect early ventilation deficits in children with CF. 

Despite the increasing popularity of LCI in monitoring CF patients with mild lung disease, it is not a 100% sensitive method in detecting ventilation defects, and other methods might be better suited for some patients. In our study, 32% of children with normal LCI results presented with perfusion deficits in MRI. 

If our results were to influence the clinical practice, MRI should be used for monitoring patients with normal LCI. On the other hand, since most patients with abnormal results of IOS and spirometry had perfusion deficits in MRI, whereas normal RV/TLC ratio in body plethysmography was in most cases associated with no perfusion deficits in MRI, in these groups of patients, MRI is less likely to provide any additional information, unless in dubious cases. An additional indication for MRI might be to target specific therapies, e.g., physiotherapy towards specific lung regions, and to monitor their results. 

## Figures and Tables

**Table 1:** Patients characteristics.

Variable	Median (Range) or Number (%)
Male/female	18/21
Diagnosed with newborn screening	15 (40)
BMI (median; range)	18.35 (13.6–34.13)
BMI z-score < −1.64 *	4 (10)
F508del homozygous	19 (49)
F508 heterozygous	15 (38)
Other mutations	3 (13)
Exacerbation (visits)	22 (25)
FEV_1_ z-score < −1.64 *	25 (29)
FEV_1_ z-score (median; range)	−0.05 (−9.06–3.71)
LCI 2.5% (median; range)	8.03 (5.1–17.39)
LCI 2.5% z-score (median; range)	4.23 (−2.95–27.16)
LCI 2.5% z-score > 1.64 *	46 (65)
Pancreatic insufficiency	34 (87)
**Medication used**	
Dornase alfa	39 (100%)
Hypertonic saline	39 (100%)
Chronic inhaled antibiotics #	13 (33%)
Modulators	0 (0%)

* During at least one visit; # Collistin or Tobramycin. Abbreviations: BMI—body mass index; FEV_1_—Forced vital capacity in the first second; LCI—Lung clearance index.

**Table 2:** Comparison of MRI perfusion score and pulmonary function tests results.

Variable	MRI Score 0	MRI Score ≥ 1	Total	Cohen’s Kappa	*p*-Value
**Multiple-breath washout test**					
LCI z-score ≤ 1.64	17	8	25	0.429	<0.001
LCI z-score > 1.64	11	35	46		
M1/M0 z-score ≤ 1.64	17	10	27	0.377	0.001
M1/M0 z-score > 1.64	11	33	44		
M2/M0 z-score ≤ 1.64	15	8	24	0.361	0.002
M2/M0 z-score > 1.64	13	35	47		
**Spirometry**					
FEV_1_ z-score ≥ −1.64	32	30	62	0.380	<0.001
FEV_1_ z-score < −1.64	0	25	25		
FVC z-score ≥−1.64	31	38	69	0.224	0.002
FVC z-score < −1.64	1	17	18		
FEF_25/75_ z-score ≥ −1.64	30	31	61	0.314	<0.001
FEF_25/75_ z-score < −1.64	2	24	26		
**Impulse oscillometry**					
X5Hz z-score ≥ −1.64	32	52	84	0.014	0.434
X5Hz z-score < −1.64	0	1	1		
X10Hz z-score ≥ −1.64	32	48	80	0.073	0.073
X10Hz z-score < −1.64	0	5	5		
Fres z-score ≤ 1.64	28	44	72	0.036	0.578
Fres z-score > 1.64	4	9	13		
**Body plethysmography**					
TLC z-score ≤ 1.64– ≥ −1.64	8	18	26	−0.086	0.443
TLC z-score < −1.64 or >1.64	21	32	53		
RV/TLC z-score ≤ 1.64	13	2	15	0.454	<0.001
RV/TLC z-score > 1.64	16	48	64		
**Single-breath carbon monoxide uptake**					
TLC z-score ≤ 1.64– ≥ −1.64	6	17	23	−0.194	0.151
TLC z-score < −1.64 or >1.64	14	17	31		
RV/TLC z-score ≤ 1.64	11	9	20	0.285	0.036
RV/TLC z-score > 1.64	9	25	34		
TLCO z-score ≥ −1.64	16	15	31	0.322	0.010
TLCO z-score < −1.64	4	19	23		

Abbreviations: M1/M0—first-moment ratio z-score; M2/M0—second-moment ratio z-score; FEV_1_ z-score—forced expiratory volume in the first-second z-score; FVC z-score—forced vital capacity z-score; FEF25/75 z-score—forced expiratory flow between 25 and 75% of FVC z-score; X5Hz z-score—respiratory reactance at 5 Hz z-score; X10Hz z-score—respiratory reactance at 10 Hz z-score; Fres z-score—resonance frequency z-score; TLC—total lung capacity z-score; RV/TLC z-score—residual volume (RV) and total lung capacity ratio z-score; TLCO z-score—transfer factor for carbon monoxide z-score.

**Table 3:** Characteristics of patients depending on their MRI perfusion score.

Variable	MRI Score			*p*-Value *
	0	1–3	>3	
**Number of visits**	36	31	20	
**Age** **(median; range)**	10 (7–17)	13 (8–18)	14 (9–18)	0.025 * (0) vs. (>3) **
**BMI** **(median; range)**	19.32 (14.47–34.13)	17.66 (13.6–23.94)	18.36 (14–21.23)	0.417 *
**F508del homozygous (%)**	13 (36%)	17 (55%)	11 (55%)	0.224 #
**F508del heterozygous (%)**	21 (58%)	10 (32%)	7(35%)	0.067 #
**PI (%)**	27 (75%)	30 (97%)	19 (95%)	0.014 # (0) vs. (1–3) ##
**Exacerbation (%)**	2 (6%)	13 (42%)	8 (40%)	0.001 # (0) vs. (1–3) ## (0) vs. (>3) ##
**Chronic** ***P. aeruginosa* infection (%) ^**	5 (14%)	7 (23%)	3 (15%)	0.614 #
**Chronic *S. aureus* infection (%) ^**	30 (83%)	30 (97%)	16 (80%)	0.135 #

* Kruskall–Wallis test with ** post-hoc Dunn–Bonferroni test; # Chi-square test and ## chi-square test with multiple comparisons corrected by Benjamini–Hochberg method ^ chronic *S. aureus and P. aeruginosa* infections were diagnosed when half of the cultures taken during the given year were positive for the given bacterial species, provided at least four cultures were taken. Abbreviations: BMI—body mass index; PI—pancreatic insufficiency.

**Table 4:** Correlations of lung function parameters with MRI perfusion score.

Variable	R Spearman	*p*-Value ^
**Multiple-breath washout**		
LCI 2.5%	0.504	<0.001
LCI z-score	0.506	<0.001
M1/M0 z-score	0.493	<0.001
M2/M0 z-score	0.347	0.049
**Spirometry**		
FEV_1_ z-score	−0.546	<0.001
FVC z-score	−0.516	<0.001
FEF_25/75_ z-score	−0.382	0.004
**Impulse oscillometry**		
X5Hz z-score	−0.394	0.003
X10Hz z-score	−0.253	0.311
AX	−0.0796	1
Fres z-score	0.0434	1
**Body plethysmography**		
TLC z-score	0.0374	1
RV z-score	0.3794	0.011
RV/TLC z-score	0.4207	0.002
**Single-breath carbon monoxide uptake**		
TLC z-score	−0.3183	0.343
RV z-score	0.013	1
RV/TLC z-score	0.2302	1
TLCO z-score	−0.3621	0.133

^ With Bonferroni correction for multiple comparisons; Abbreviations: LCI 2.5%—lung clearance index at 2.5% stopping point; LCI z-score—lung clearance index z-score; M1/M0—first-moment ratio z-score; M2/M0—second-moment ratio z-score; FEV_1_ z-score—forced expiratory volume in the first-second z-score; FVC z-score—forced vital capacity z-score; FEF25/75 z-score—forced expiratory flow between 25 and 75% of FVC z-score; AX—area of reactance, RV—residual volume; RV z-score—residual volume z-score; X5Hz z-score—respiratory reactance at 5 Hz z-score; X10Hz z-score—respiratory reactance at 10 Hz z-score; Fres z-score—resonance frequency z-score; TLC z-score—total lung capacity z-score; RV/TLC z-score—residual volume (RV) and total lung capacity ratio z-score; TLCO z-score—transfer factor for carbon monoxide z-score.

## Data Availability

The datasets used and analyzed during the current study are available from the corresponding author upon reasonable request.
